# Epigenetics in asthma: implications for treatable traits

**DOI:** 10.3389/falgy.2026.1929559

**Published:** 2026-09-07

**Authors:** Vasiliki Apollonatou, Argyro Vrouvaki, Marina Moustaka Christodoulou, Athanasios Fengas, Stelios Loukides, Evangelia Fouka

**Affiliations:** 1 2nd Respiratory Medicine Department, Medical School, National and Kapodistrian University of Athens, Attikon University Hospital, Athens, Greece; 2 Department of Pneumonology, 401 General Military Hospital of Athens, Athens, Greece

**Keywords:** asthma, DNA methylation, epigenetics, histone modifications, long non-coding RNAs, microRNAs, treatable traits

## Abstract

Epigenetic mechanisms refer to heritable and potentially reversible modifications that regulate chromatin architecture and gene expression without altering the underlying DNA sequence. In asthma, these mechanisms provide a molecular interface between genetic susceptibility, environmental exposures, and immune dysregulation, contributing to disease heterogeneity and treatment response. The treatable traits paradigm, which focuses on the identification of specific modifiable biological and clinical characteristics, offers a clinically relevant framework for linking epigenetic changes to distinct molecular endotypes, enhancing mechanistic stratification beyond traditional phenotyping, thereby supporting precision medicine approaches. Emerging evidence suggests that epigenetic signatures have considerable potential as biomarkers for inflammatory endotyping and therapeutic stratification, although further validation is required before routine clinical implementation. In this mini review we will discuss epigenetic mechanisms involved in the development and maintenance of asthma processes, such as DNA methylation, histone modifications, and noncoding RNAs, focusing on their relevance to key treatable traits, including type 2 inflammation, corticosteroid responsiveness, airway remodeling, and exposure-driven disease.

## 1 Introduction

Asthma is a biologically heterogeneous airway disease affecting both children and adults worldwide, characterized by chronic airway inflammation, increased hyperresponsiveness, structural remodeling, and variable responses to therapy (1). Early life environmental exposures, including allergens, air pollutants, infections, and dietary factors, can induce persistent alterations in immune regulation, thereby influencing disease susceptibility, airway development, and long-term lung function trajectories (2). In recent years, several studies have identified various genetic variants that play a significant role in asthma susceptibility (3). However, these effects cannot be fully explained by genetic variation alone, highlighting the importance of epigenetic mechanisms in asthma pathogenesis (4, 5). Epigenetics in asthma refers to heritable and potentially reversible modifications of chromatin architecture and gene expression that occur without alterations in the DNA sequence (6, 7). Epigenetic mechanisms, such as DNA methylation, histone modifications, and non-coding RNAs (ncRNAs), regulate transcriptional programs in airway epithelial, smooth muscle, and immune cells, providing a critical link between environmental exposures and immune and inflammatory pathways that contribute to disease pathogenesis and progression (8, 9). Beyond the aforementioned mechanisms, epitranscriptomic mechanisms, particularly N6-methyladenosine (m6A) RNA methylation play an important role in epigenetic regulation in asthma. M6A is the most abundant internal RNA modification in eukaryotic mRNA and regulates RNA splicing, stability, export, and translation through the coordinated actions of “writers”, “erasers”, and “readers”. Emerging evidence suggests that dysregulated m6A signaling contributes to immune cell differentiation, airway inflammation, and asthma endotypes, highlighting its potential role in biomarker development and precision medicine (10, 11). Within this context, the treatable traits paradigm, which focuses on the identification of specific modifiable biological and clinical characteristics, may provide a valuable clinical framework linking epigenetic changes to distinct molecular endotypes, thereby enhancing mechanistic stratification beyond traditional phenotyping (12, 13). As epigenetic modifications are dynamic and potentially reversible, they represent attractive candidates for biomarker development and therapeutic intervention. Moreover, unlike genetic variants, epigenetic alterations may reflect current disease activity, environmental exposures, and treatment responsiveness, making them particularly relevant within the treatable traits framework.

This narrative mini-review is based on English-language literature searches of MEDLINE through April 2026, summarizing the current evidence on epigenetic mechanisms in asthma. In this mini-review, we aim to synthesize epigenetic mechanisms that map onto clinically actionable asthma treatable traits and highlight how epigenetic mechanisms may facilitate implementation of precision medicine through clinically actionable treatable traits. Rather than providing a comprehensive overview of asthma epigenetics, this review specifically highlights mechanisms with potential clinical relevance for inflammatory endotyping, biomarker development, and precision treatment within the treatable traits paradigm.

## 2 Epigenetic mechanisms in asthma

### 2.1 DNA methylation

DNA methylation is the most extensively studied epigenetic mechanism associated with asthma. It affects gene expression by inhibiting transcription, causing gene silence (14). During methylation, a methyl group is added to the cytosine ring, forming 5-methylcytosine (5-mC) (15). 5-mC block enzymes are responsible for DNA transcription, so gene expression is not achieved. The enzymes that comprise methyl groups are known as DNA methyltransferases (DNMTs). There are three major classes of DNMTs: DNMT1, DNMT3a, and DNMT3b. DNMT1 is responsible for maintaining the methylation of parent DNA, whereas DNMT3a and DNMT3b cause *de novo* methylation (16).

DNA methylation has been proposed to act as an interface between the environment and the regulation of gene expression. Environmental exposures, such as cigarette smoke, particulate matter (especially PM_2.5_), and infections, induce methylation shifts that contribute to asthma-related epigenetic alterations (17). More specifically, air pollution has been linked to lung pathology, including asthma, and multiple studies have proven a direct impact on DNA methylation (18). PM_2.5_ seems to alter DNA methylation in the very early stages (*in utero*) (19), and murine studies have shown that exposure to this matter increases DNA methylation at the IFN*γ* promoter in CD4^+^ T cells, thus shifting towards a T2 subtype of asthma (20). Moreover, exposure to air pollution seems to play a significant role in the balance between DNA methylation and demethylation through increased methylation levels at the promoter of the DNA demethylating enzyme ten-eleven translocation 1 (*TET1)* (21). Infections have also been implicated in DNA methylation. They promote the release of oxidants and proinflammatory mediators in the airways, which exert epigenetic modifications on the transcriptional programs of cytokines, triggering methylation (22).

Collectively, DNA methylation represents the best-characterized epigenetic mechanism in asthma and provides a molecular interface between environmental exposures and immune dysregulation. Nevertheless, most available evidence derives from association studies, and causal relationships remain to be established.

### 2.2 Histone modifications

Another important epigenetic mechanism for enhancing gene transcription is histone remodeling through acetylation. Histones are proteins that pack DNA to fit in the nucleus, specifically in the nucleosomes (23). Responsible for histone acetylation are enzymes called histone acetyltransferases (HATs). In addition, there are enzymes called histone deacetylases (HDACs) that remove acetyl groups from histones, causing repacking of chromatin and thus reducing gene expression (24). The balance between HATs and HDACs is responsible for the acetylation of histones and, therefore, for gene expression or silencing.

In patients with asthma, this balance is altered, with increased HAT levels, resulting in high gene expression of inflammation in the brochi (25). Similarly, alveolar macrophages seem to have reduced HDAC expression, causing inflammatory gene expression in patients with asthma (26). In particular, HDAC1 is responsible for airway remodeling and epithelial repair, and studies have shown that HDAC1 levels are increased in severe asthma compared to mild asthma (27). Defective HDAC2 activity is relevant to corticosteroid-resistant severe asthma phenotypes (28). Moreover, histone acetylation also represents an important interface through which environmental and dietary factors modulate immune responses, suggesting that lifestyle-related interventions may influence asthma susceptibility through epigenetic mechanisms. Recent evidence indicates that histone acetylation regulates gene expression in a cell-specific manner across airway epithelial cells, CD4⁺ T lymphocytes, macrophages, fibroblasts, and airway smooth muscle cells, thereby influencing multiple components of asthma pathobiology (29). Beyond therapeutic implications, histone acetylation signatures may also contribute to future biomarker development and molecular endotyping, although technical standardization and validation remain necessary before clinical implementation. Beside acetylation, H3K27 trimethylation is increased in airway epithelial cells of patients with asthma, thus inhibiting the expression of immune genes, including IL-4 and IL-13, causing immune tolerance loss and maintaining the chronic inflammatory phase (30). Although histone acetylation remains the best-characterized histone modification in asthma, accumulating evidence suggests that other histone modifications, such as histone methylation, phosphorylation, ubiquitination, SUMOylation and ADP-ribosylation, also participate in immune-cell differentiation, inflammatory gene regulation and airway remodeling, contribute to the dynamic regulation of gene expression by influencing chromatin structure and the recruitment of transcriptional regulators, highlighting the complexity of the epigenetic landscape in asthma (31–33). Overall, unlike DNA methylation, histone modifications are highly dynamic and pharmacologically reversible, making them particularly attractive therapeutic targets. The association between altered HDAC activity and corticosteroid resistance further supports their clinical relevance, particularly in severe asthma.

### 2.3 MicroRNAs and long non-coding RNAs

Non-coding RNAs, including microRNAs (miRNAs) and long non-coding RNAs (lncRNAs), regulate post-transcriptional gene expression by controlling the translation of approximately 60% of mRNAs (34) and have been increasingly implicated in asthma pathobiology. MiRNAs modulate cytokine signaling, immune cell differentiation, and airway remodeling, promoting T2 polarization and allergic inflammation (35). Several miRNAs have consistently emerged as regulators of asthma pathobiology, with some promoting type 2 inflammation (e.g., miR-21, miR-126 and miR-19), whereas others appear to exert anti-inflammatory effects (e.g., miR-223). Overexpression of specific miRNAs, such as miR-21 and miR-126, correlates with elevated IL-13 levels, enhanced immune cell migration in bronchial asthma (36), and is linked to steroid insensitivity in asthmatic children (37), while miR-19 upregulation promotes IL-5 and IL-13 production in airway T cells (38). MiR-21 and miR-126 are upregulated in asthmatic patients compared to control, especially in patients who do not receive treatment (36), and miR-21 seems to contribute to eosinophilic production and proliferation, while miR-223 has the opposite result (39). In general, miR-21 is the best-studied miRNA in asthma, with significant correlations with steroid resistance, thus making it a possible biomarker for the evaluation of treatment response (37).

LncRNAs are RNA transcripts that are more than 200 nucleotides in length (40). They regulate gene expression through miRNA sponging, transcriptional scaffolding and chromatin modulation (41). In recent years, several studies have been conducted to find a correlation between specific lncRNAs and asthma. Specifically, LncRNA-MEG3 functions as a competing endogenous RNA to modify the regulatory T cell (Treg)/T helper 17 (Th17) balance in patients with asthma by targeting microRNA-17/ROR*γ*t (42). Another study showed that increased expression of lncRNA signatures in blood eosinophils promotes T2 inflammation via GATA3 in Patients With Eosinophilic asthma (43). Ma et al. found that seven lncRNAs were downregulated in the peripheral blood of asthmatic patients compared to healthy subjects, indicating that lncRNAs could serve as potential biomarkers for asthma diagnosis (44).

### 2.4 RNA modifications

Beyond DNA and histone modifications, chemical modifications of RNA have emerged as an additional layer of epigenetic regulation. Among these, m6A is the most abundant internal modification of messenger RNA and influences RNA splicing, nuclear export, stability and translation through the coordinated activity of methyltransferases (“writers”), demethylases (“erasers”), and m6A-binding proteins (“readers”). Recent evidence indicates that dysregulated m6A signaling contributes to asthma pathogenesis by regulating inflammatory cell differentiation, epithelial responses, cytokine expression and airway remodeling. Altered expression of m6A regulators has been associated with distinct inflammatory asthma endotypes and immune-cell infiltration patterns, suggesting that RNA methylation represents a promising biomarker and therapeutic target within precision medicine approaches (11, 45). Because of its dynamic and reversible nature, m6A is increasingly recognized as an important component of the asthma epigenetic landscape.

## 3 Epigenetics mapped to treatable traits

Treatable traits refer to a precision medicine approach (46) in which the patient undergoes a multisystemic assessment to identify clinically important problems that are treatable and called traits (47). A treatable trait is clinically important, recognizable, and responsive to specific therapy (48). These traits are categorized into three domains: pulmonary issues, comorbidities, and behavioral factors that contribute to the disease burden. In asthma, some important treatable traits include (but are not limited to) inhaler device technique and adherence, type 2 (T2) inflammation, airflow limitation, diet, and smoking. Literature search has shown that some of these traits are affected by epigenetic changes (Table 1). Thus, in the following sections, we will analyze the implications of epigenetic modifications for each trait separately.

**Table 1:** Treatable traits of asthma and related epigenetic mechanisms.

Treatable trait	Epigenetic mechanism	Key targets	Clinical implication
T2 inflammation	DNA methylation	IL-4	Eosinophilia
		IL-13	FeNO
		*EPX*	IgE
		TIGIT	
Steroid resistance	Histone modifications	HDAC2	Reduced corticosteroid response
		*VNN1*	
Airway remodeling	Histone acetylation	HDAC8	Fixed airflow limitation
	miRNAs	miR-23b	
Exposure-driven asthma	DNA methylation	*TET1*	Pollution-related disease
		*NOS3*	
		*FOXP3*	
Smoking-related asthma	DNA methylation	*AHRR*	Steroid resistance
		*CYP1A1*	
Obesity-associated asthma	DNA methylation	Aging-related pathways	Increased severity

T2, Type 2; IL-4, Interleukin 4; IL-13, Interleukin 13; *EPX*, Eosinophil peroxidase; TIGIT, T cell immunoreceptor with Ig and ITIM domains; FeNO, Fractional exhaled nitric oxide; IgE, Immunoglobulin E; HDAC2, Histone deacetylase 2; *VNN1*, Vanin-1; HDAC8, Histone deacetylase 8; miRNAs, MicroRNAs; miR-23b, MicroRNA-23b; *TET1*, Ten-eleven translocation 1; *NOS3*, Nitric oxide synthetase 3; *FOXP3*, Forkhead box P3; *AHRR*, Aryl-hydrocarbon receptor repressor; *CYP1A1*, Cytochrome P450 family 1 subfamily A member 1.

### 3.1 T2 inflammation

T2 inflammation is the most thoroughly characterized treatable trait in asthma and is characterized by eosinophilia, elevated fractional exhaled nitric oxide (FeNO), increased immunoglobulin E (IgE), and activation of the IL-4/IL-5/IL-13 axis. Emerging evidence suggests that epigenetic regulation plays a role in both the establishment and persistence of T2 inflammatory endotypes (49). Van Asselt et al (50) invested the relationship between DNA methylation and clinical markers of asthma and found 270 CpGs that were associated with clinical markers of asthma, with most associations being about IgE, blood eosinophils and airway inflammation, suggesting that DNA methylation profile could be a stable biomarker for asthma. In addition to DNA methylation, RNA methylation, particularly m6A, has been implicated in severe asthma. M6A modification is the most prevalent form of RNA modification in eukaryotic and viral RNA (51). Distinct m6A regulator–mediated RNA methylation modification patterns are associated with different immune microenvironment infiltration profiles and correlate directly with T2 inflammation. More specifically, m6A RNA methylation regulates the expression of genes involved in T2 inflammation, eosinophil recruitment, T helper 2 cell differentiation, and airway remodeling, thereby contributing to the development and progression of asthma. Key m6A regulators, including methyltransferase-like 3 and 14 (METTL3, METTL14) fat mass and obesity-associated protein (FTO), alkB homolog 5 (ALKBH5) and YT521-B homology (YTH) domain-containing proteins, modulate the stability and translation of cytokine- and immune-related mRNAs, influencing inflammatory phenotypes and representing potential biomarkers and therapeutic targets for distinct asthma subtypes (11). Han et al. (45) demonstrated that m6A RNA methylation suppresses allergic airway inflammation by maintaining macrophage homeostasis through the METTL3-pentraxin 3 (PTX3) signaling axis. Specifically, METTL3-mediated m6A modification enhances the stability and expression of PTX3 mRNA, promoting anti-inflammatory macrophage function, whereas loss of METTL3 reduces PTX3 expression, disrupts macrophage homeostasis, and exacerbates eosinophilic airway inflammation and allergic asthma (45). m6A RNA methylation plays also an important role in regulating the expression of pathogenic genes, meaning that abnormal m6A modification can affect RNA splicing, translocation, and translation, resulting in the occurrence of asthma (52). Sun et al (52) found that 16 m6A regulators were altered in the severe asthma group compared with healthy subjects and that eosinophil infiltration was regulated by many m6A regulators, among which there was a significant positive correlation with YT521-B homology m6A RNA-binding protein 3 (YTHDF3) and a significant negative correlation with eukaryotic translation initiation factor 3 subunit B (EIF3B). Co-methylation analyses have identified four modules, two of which were linked to asthma severity through airway remodeling pathways (TNF- and TGF-*β*1–regulated modules), one associated with eosinophilia, and another associated with increased FeNO (53). Notably, methylation patterns were not altered by ICS/LABA treatment *in vitro*, suggesting that these signatures may reflect disease-driving processes rather than treatment-induced changes. The identification of four methylation modules corresponding to distinct biological programs raises the possibility of defining four epigenetically informed endotypes.

DNA hypomethylation of specific genes can trigger T2 and eosinophilic responses, resulting in asthma and allergy phenotypes. Specifically, Cardenas et al. showed that differentially methylated regions were associated with genes implicated in allergic asthma, T2 activation, and eosinophilia [Eosinophil peroxidase (*EPX)*, *IL-4, IL-13*] in Epigenome-Wide Association studies (EWAS) of blood (54). This study showed that allergic asthma and elevated biomarkers of allergic disease, such as FeNO and IgE, are associated with multiple differentially methylated CpGs and regions of genes that may alter the structure and function of epithelial cells and genes implicated in allergic asthma, T2 activation, and eosinophilia. Moreover, a meta-analysis of two EWAS of asthma using DNA from white blood cells from 1,136 Latino children and young adults showed that T cell immunoreceptor with Ig and ITIM domains (TIGIT) methylation can be used as a therapeutic target in T2 asthma (55). Collectively, these findings suggest that epigenetic profiling may complement established biomarkers such as blood eosinophils and FeNO by identifying molecularly distinct T2 endotypes beyond conventional inflammatory markers.

### 3.2 Steroid responsiveness vs. resistance

There is considerable heterogeneity in the response to corticosteroid treatment in patients with asthma, and epigenetic factors may play an important role in this process. Corticosteroids are the first-line treatment for patients with asthma. They decrease inflammatory process in the airways by suppressing the activation of proinflammatory transcription factors, such as NF-*κ*B (56). They also recruit HDAC2 enzymes, resulting in the inhibition of the acetylation of active inflammatory genes (56). Resistance to corticosteroids, as seen in some patients with asthma, may be due to a defect in HDAC2 activity. Therefore, a new therapeutic strategy could be activators of these enzymes. Theophylline is an example of the first drug that has been shown to have activating properties over HDACs (57). Mishra et al (58) showed that theophylline has the potential to induce a response against steroid resistance in patients with severe asthma by increasing HDAC2 mRNA expression. Corticosteroid resistance could also be explained by differences in gene expression. Xiao et al (59) showed that changes in vanin-1 (*VNN1*) mRNA expression were associated with systemic corticosteroid treatment response in children with acute asthma, and that *VNN1* was required for an optimal response to corticosteroid treatment in an experimental asthma model. Furthermore, a CpG site within the *VNN1* promoter was differentially methylated between good and poor treatment response groups, and methylation at this site correlated with *VNN1* mRNA expression (59). Another possible mechanism of corticosteroid resistance is exposure to smoke. A study was conducted to investigate the relevance of smoking-associated DNA methylation changes in the lower airways by examining the nasal epithelium in patients with asthma who were current or ex-smokers (60). A total of 809 genes and 18,814 CpG sites were differentially associated with current smoking in the nose of the participants. A *cis*-eQTM analysis uncovered 171 CpG sites with a methylation status associated with smoking-related gene expression, including aryl-hydrocarbon receptor repressor (*AHRR)*, aldehyde dehydrogenase 3 family member A1 (*ALDH3A1)*, cytochrome P450 family 1 subfamily A member 1 (*CYP1A1)*, and cytochrome P450 family 1 subfamily B member 1 (*CYP1B1)*. The methylation status of CpG sites altered by current smoking was reversed after 1 year of smoking cessation, confirming that current smoking alters epigenetic patterns and affects gene expression in the nasal epithelium of patients with asthma. These observations support the concept that corticosteroid responsiveness represents not only a pharmacological phenotype but also an epigenetically regulated treatable trait.

### 3.3 Airway remodeling

Airway smooth muscles (ASM) are implicated in a variety of functions in the initiation and maintenance of asthma processes, such as contraction, inflammation, and gradual airway structural changes leading to remodeling. Compared with inflammatory biomarkers, epigenetic regulation of airway remodeling remains less well characterized, however it represents an attractive avenue for identifying patients at risk of irreversible airflow limitation. Epigenetic changes in ASM could shift the airways to an abnormal response to allergens, thus developing inflammation and subsequently remodeling and fixed airway obstruction. Histone modifications regulate ASM remodeling in asthma (61). HDAC8 can regulate the cytoskeletal dynamics of ASM (62), and histone arginine modifications decrease ASM cell activity (63). Previous research has shown that decreased HDAC activity, increased HAT activity, and histone 3 panacetylation have been found in the bronchial biopsies of patients with asthma, and acetylation was shown to affect the expression of multiple genes associated with airway inflammation and airway remodeling (25). Moreover, studies have demonstrated that miR-23b inhibits airway smooth muscle proliferation (64), a factor that could act as a potential therapeutic target for irreversible airway remodeling (65).

### 3.4 Exposure-driven asthma

**Figure 1:**
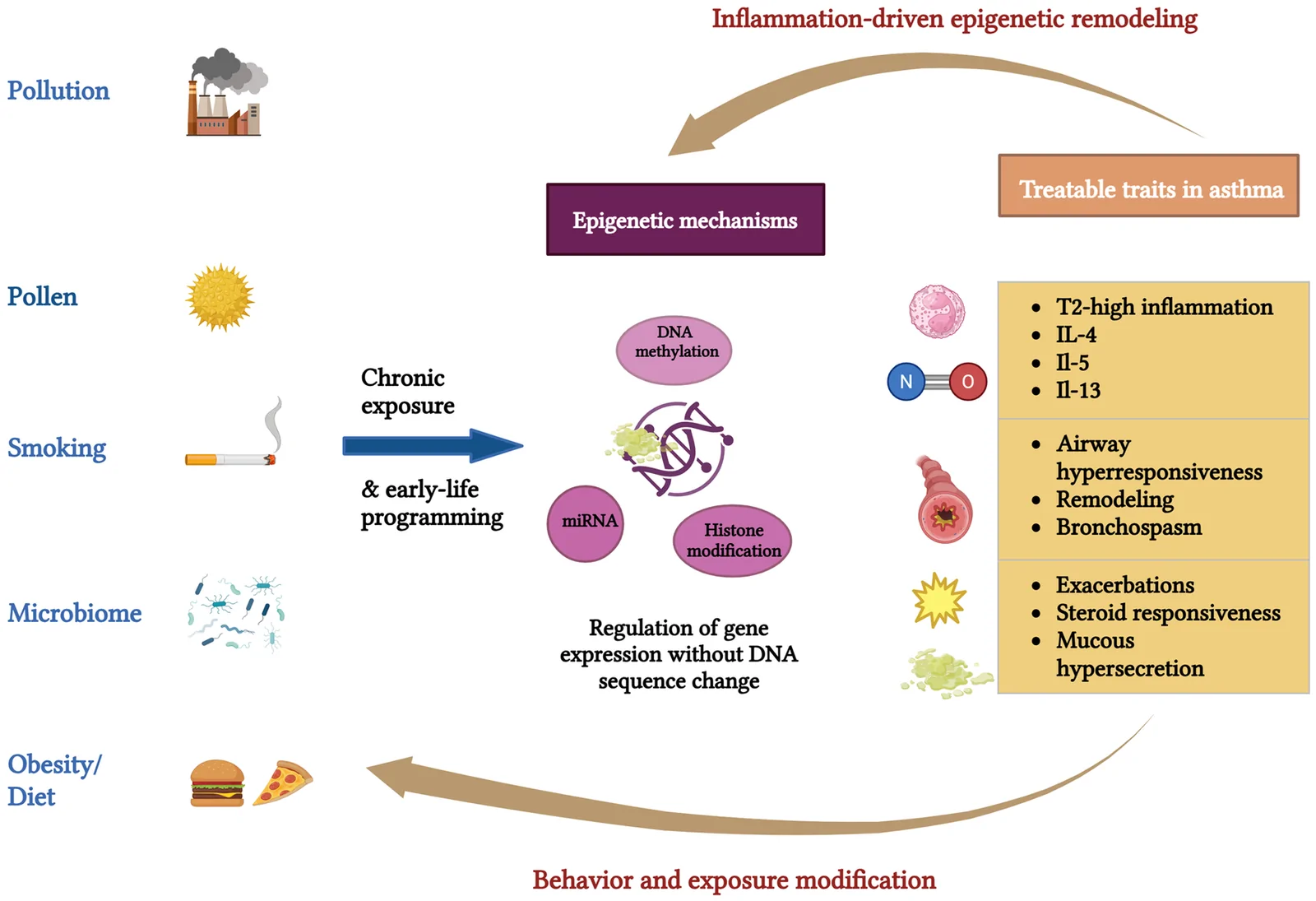
Environmental exposures shape asthma treatable traits through epigenetic mechanisms. Environmental exposures, including air pollution, allergens, cigarette smoke, alterations of the microbiome, and obesity-related dietary factors, induce epigenetic modifications through chronic exposure and early-life programming. These changes, including DNA methylation, histone modifications, and non-coding RNA (miRNA)-mediated regulation, alter gene expression without modifying the DNA sequence, thereby influencing key biological pathways involved in asthma. Epigenetic remodeling contributes to the development of clinically relevant treatable traits, including type 2 (T2)-high inflammation, airway hyperresponsiveness, airway remodeling, mucus hypersecretion, corticosteroid responsiveness, and exacerbation susceptibility. In turn, persistent airway inflammation may further promote epigenetic remodeling, whereas modification of environmental exposures and lifestyle factors may attenuate these processes, highlighting the dynamic and potentially reversible nature of epigenetic regulation in asthma. Created in BioRender. Fouka, E. (2026) https://BioRender.com/vw0uqic, licensed under CC BY 4.0.

As previously discussed, environmental exposure plays a significant role in asthma-related epigenetic alterations (17). Such exposures include allergens, environmental pollution, cigarette smoke, and infections, especially viral. Several allergens, such as house dust mites (HDM), pet dander, mold, and pollen, have been identified as triggers of allergic asthma, and recent advancements in molecular biology have highlighted the crucial role of epigenetic mechanisms in immune gene expression (66) (Figure 1). Acetylation of the promoter regions of allergy-related immune genes has been identified as a significant risk factor for sensitization to inhaled allergens (67). Moreover, histone acetylation regulates cytokines involved in allergic diseases, and T2 differentiation is accompanied by dynamic alterations in histone acetylation at cytokine gene loci (66). In addition to histone acetylation, miRNAs are also involved in the inflammatory process. Exposure to dust mite antigens activates toll-like receptor 4 (TLR4) and increases the expression of miRNAs, including miRNA-16, miRNA-21, and miRNA-126 (68).

Outdoor pollution, especially diesel exhaust particles (DEP), which are a significant contributor to particulate matter in traffic-related air pollution, is known to exacerbate asthma phenotypes. Zhu et al (69) studied nasal epithelium DNA methylation and gene expression in children with severe and non-severe asthma. They identified 816 differentially methylated CpG positions (DMPs) and 10 differentially methylated regions (DMRs) associated with asthma severity. Specifically, three DMPs showed discriminatory ability for severe asthma, and six DMPs were associated with asthma, allergic asthma, IgE, and FeNO, while 27 DMPs were associated with traffic pollution or secondhand smoke. Ambient air pollution exposure can also cause epigenetic changes by increasing the DNA methylation of transcription factors. In particular, Nadeau et al (70) examined 2 groups of asthmatic and non-asthmatic children who were exposed to different air pollutant levels and found that children who lived in a more polluted place often showed hypermethylation of forkhead box P3 transcription factor (*FOXP3*), which impairs the activity of regulatory T cells and increases asthma morbidity. Another study showed that exposure to traffic-related air pollution was associated with higher levels of FeNO and lower levels of DNA methylation in the promoter regions of the nitric oxide synthetase (NOS) 3 gene, indicating that DNA methylation of the *NOS3* gene could be an important epigenetic mechanism in physiological responses to exposure to traffic-related air pollution in children with asthma (71). Although not yet a clinical biomarker, *NOS3* methylation changes show promise as an environment-linked molecular indicator of airway inflammation mechanisms.

Another significant environmental exposure contributing to asthma development is tobacco smoke. Exposure to tobacco smoke enhances sensitization to various allergens, and the earlier the exposure, the greater the risk (72). Epigenetic alterations due to tobacco smoke are caused via induction of oxidative stress, specifically through the disruption of the HAT/HDAC balance in different immune cells of the airways (22). Ito et al (73) compared biopsies and bronchoalveolar lavage alveolar macrophages from nonsmokers and smokers and proved that exposure to tobacco smoke suppressed HDAC2 expression and overall HDAC activity and enhanced the expression of inflammatory mediators. Moreover, there was a significant attenuation of dexamethasone-induced inhibition of cytokine release, causing resistance to steroids. In addition to HAT/HDAC homeostasis, smoking can affect epigenetic actions by altering DNA methylation in gene promoters and/or regulatory sequences (22). DNA methylation may also be an epigenetic mechanism that could explain the lifelong effect of exposure to smoking *in utero* on asthma risk (74). In fact, prenatal maternal smoking is associated with low birth weight, neurological disorders, and asthma in exposed children. DNA methylation can serve as a biomarker for prenatal tobacco exposure. Developmentally patterned methylation changes have been described in loci relevant to airway biology and remodeling. For example, methylation of the *SMAD* family member 3 promoter region, assessed in epithelial mucosal cells at birth and preschool age, was associated with asthma and influenced by both genetic and environmental factors, supporting a developmental window in which remodeling-related pathways may be epigenetically tuned (75). These findings support the concept that early life methylation signatures can serve as mechanistic intermediates linking exposure to the expression of diseases later in life. Prenatal smoke exposure is one of the strongest examples of exposure-associated methylation with potential persistence. Cord blood studies have identified numerous loci responding to maternal smoking during pregnancy, with signals persisting into later childhood, providing mechanistic insight into how a major modifiable exposure shapes downstream respiratory outcomes (76). Another study examined polymethylation scores derived from cord blood CpGs, offering a non-invasive biomarker of past maternal smoke exposure and enabling longitudinal and interventional study designs that avoid repeated blood sampling (77). These approaches operationalize “exposure burden” as a quantifiable trait that can be incorporated into risk stratification and prevention strategies for various diseases.

Respiratory infections, especially viral infections, in early life are strongly associated with the development of childhood asthma (78). It is not fully understood if these infections cause direct airway damage or whether they reveal underlying host susceptibility. Changes in DNA methylation patterns have been reported following viral infections and may persist long after viral clearance, potentially contributing to long-term disease susceptibility (79). Pishedda et al (80) examined epigenetic changes in children with respiratory syncytial virus (RSV) infection and found that individuals who later developed asthma showed more alterations in DNA methylation biomarkers, especially hypomethylation patterns, suggesting that RSV infection could initiate asthma-related epigenetic alterations. Additionally, human rhinovirus (HRV) infections are known to induce specific DNA methylation changes in immune function-related genes, such as *CXCR4* and *HLA-H*, proving that HRV may contribute to the progression of allergic diseases through epigenetic modifications (81). Taken together, these studies indicate that epigenetic modifications may function as molecular archives of cumulative environmental exposure, linking external insults with long-term alterations in asthma susceptibility and severity.

While considerable attention has focused on the detrimental effects of environmental exposures, increasing evidence indicates that certain early-life exposures exert protective effects against asthma development. The “hygiene hypothesis”, subsequently expanded into the biodiversity and farm-effect concepts, proposes that exposure to diverse microbial environments during early life promotes immune tolerance and reduces the risk of allergic diseases (82). Emerging evidence suggests that these protective effects are mediated, at least in part, through epigenetic mechanisms, including DNA methylation and histone modifications affecting regulatory T-cell function and immune maturation. Farm-associated microbial exposure has been linked to epigenetic regulation of genes involved in innate and adaptive immunity, supporting the concept that environmental influences may shape asthma susceptibility through both harmful and protective epigenetic programming (83). These observations further emphasize that epigenetic regulation reflects cumulative environmental experience rather than exclusively harmful exposures, reinforcing its potential value for exposure-related treatable traits and preventive strategies. Protective microbial exposures may also influence asthma susceptibility through modulation of the host microbiome. Traditional farming environments are characterized by increased microbial diversity, including environmental bacteria such as *Acinetobacter lwoffii*, which have been shown experimentally to attenuate allergic airway inflammation by promoting immune tolerance (84).

### 3.5 Diet/obesity-related asthma

In recent years, increasing attention has been focused on the role of diet as a crucial modulator of immune development and respiratory health. The human gastrointestinal tract harbors a diverse microbial community that contributes to nutrient metabolism, epithelial barrier integrity, and the regulation of local and systemic immune responses (85). Communication between the gut and the lung occurs through lymphatic circulation, hematogenous dissemination, and microaspiration, forming a bidirectional regulatory network known as the gut-lung axis (86). Recent studies have shown that alterations in gut microbial composition may be associated with asthma susceptibility, phenotypic expression, and disease progression (87). Importantly, alterations in the gut microbiome are influenced not only by dietary factors but also by environmental microbial exposures encountered during early life, providing an additional mechanism through which farm-associated environments may confer protection against asthma. An imbalance in the microbiota disrupts immune homeostasis, enhances T2 and Th17 polarization, and promotes airway hyperresponsiveness and inflammation. Dietary factors play an important role in shaping the gut microbiota composition, thereby influencing the gut-lung axis. Short-chain fatty acids (SCFAs) are key mediators of these protective effects. SCFAs signal through G protein-coupled receptors, including GPR43 and GPR41, expressed on immune cells such as eosinophils, dendritic cells, and Treg cells. Activation of these receptors suppresses eosinophil chemotaxis and degranulation, reducing T2 inflammation in the airway (88). Moreover, SCFAs function as HDAC inhibitors, thus exerting epigenetic control over immune gene expression by reducing the transcription of IL-5 and IL-13 while enhancing acetylation at regulatory loci associated with immune tolerance (89, 90). Studies in humans have also demonstrated that microbiota-derived SCFAs, particularly butyrate and propionate, enhance the differentiation and suppressive capacity of Treg cells by promoting histone acetylation at Treg associated loci, including *FOXP3*, through epigenetic modulation of chromatin accessibility and HDAC inhibition (91, 92). In contrast, high-fat diets during early life have been demonstrated to induce epigenetic changes at genome-wide level and in gene-specific loci, such as obesity, insulin resistance, and type 2 diabetes-related (93, 94), and to regulate the expression of certain miRNAs related to obesity and lipid metabolism (95). Polyunsaturated fatty acids (PUFAs) modulate the immune system by regulating cytokine production and have been implicated in the risk of allergic diseases (96). Omega-3 (*ω*-3) PUFAs are epigenetic modifiers that increase H3 acetylation in the promoters of *FOXP3*, IL-10 receptor subunit alpha, and IL-17 receptor genes, suggesting a protective role against asthma (97). In addition to histone modifications, *ω*-3 PUFAs can also modify DNA methylation levels in genes encoding IFN-*γ* and IL-13, suggesting an impact on Th1/Th2 balance (98). Conversely, *ω*-6 PUFAs is generally considered proinflammatory and is associated with an increased risk of asthma (99).

Obesity is a major risk factor for asthma and is associated with a higher incidence, greater disease severity, more frequent exacerbations, reduced responsiveness to conventional therapies, and poorer health outcomes (100). In addition to DNA methylation and histone modifications, microRNAs have recently emerged as important regulators of obesity-associated asthma. Plasma extracellular vesicle-associated miRNA signatures have been shown to distinguish obesity-associated low type 2 asthma from non-obese low type 2 asthma, reflecting distinct inflammatory and metabolic pathways. In particular, enrichment of the miR-17-92 and miR-106a-363 clusters has been associated with cytokine signaling, metabolic regulation, systemic inflammation, and lung function parameters, suggesting that circulating miRNAs may serve as biomarkers for molecular endotyping and future precision medicine approaches in obesity-associated asthma (101). These findings further support the concept that obesity-associated asthma possesses a distinct epigenetic signature involving DNA methylation, histone modifications and non-coding RNAs, reinforcing its characterization as a biologically distinct asthma endotype. Another study examined the role of epigenetics in obesity-related asthma and found that accelerated biological aging and DNA methylation modifications mediate the association between obesity and asthma (102). Current evidence suggests that obesity-related epigenetic alterations may contribute to a distinct asthma endotype characterized by reduced treatment responsiveness and accelerated biological aging, although mechanistic evidence remains limited and further studies are needed.

## 4 Future directions and clinical translation

Future research in asthma epigenetics should focus on understanding the complex interactions between genetic susceptibility, environmental exposures, and disease evolution. A major challenge remains the translation of epigenetic discoveries into clinically applicable biomarkers. Although numerous epigenetic biomarkers have been associated with asthma phenotypes and treatable traits, most remain at the discovery or early validation stage. At present, they should be considered complementary rather than alternative biomarkers to established clinical indicators such as blood eosinophils, FeNO, total IgE and sputum eosinophils. Prospective multicentre studies are required to determine their incremental clinical value and cost-effectiveness before routine implementation (103). Several important obstacles must be overcome before epigenetic biomarkers can be incorporated into routine clinical practice. These include tissue-specific variability, limited reproducibility across independent cohorts, lack of standardized analytical pipelines, and the predominance of cross-sectional rather than longitudinal studies. Addressing these limitations will be essential for validating epigenetic signatures as reliable tools for patient stratification and treatment selection. Several studies have already identified epigenetic biomarkers associated with asthma treatable traits and treatment responses (Table 2).

**Table 2:** Epigenetic biomarkers associated with treatable traits and clinical implications in asthma.

Biomarker	Sample source	Associated trait	Potential clinical use
Nasal methylome	Nasal epithelium	T2 inflammation	Endotyping
miR-26	Blood	Eosinophilic asthma	Treatment response
	Sputum		
miR-126	Blood	Allergic asthma	Disease activity
	Sputum		
*VNN1* methylation	Blood	Steroid response	Predict response
*FOXP3* methylation	Blood	Pollution-associated asthma	Risk stratification
m6A signatures	Blood	Severe asthma	Precision endotyping

T2, Type 2; miR-26, MicroRNA-26; miR-126, MicroRNA-126; *VNN1*, Vanin-1; *FOXP3*, Forkhead box P3; m6A, N6 methyladenosine.

Xue Zhang et al (104) showed that profiled nasal epithelial DNA methylation in children and identified signatures predictive of asthma control and exacerbation risk. Moreover, in an attempt to identify more biomarkers, extracellular vesicles (EVs) can be employed due to the fact that they are heterogeneous membranous nanoparticles secreted from every cell (105), carrying a multitude of intracellular bioactive molecules between cells, thus playing a key role in intracellular communication (106). EVs, including exosomes and microvesicles, have emerged as important mediators of epigenetic communication in asthma by transporting biologically active cargo, including miRNAs, lncRNAs, mRNAs, proteins and lipids, between structural and immune cells (107). Through this intercellular transfer, EVs influence epithelial integrity, airway remodeling, immune-cell polarization and inflammatory signaling. Because EV cargo reflects the physiological and pathological state of the parent cell, EV-associated non-coding RNAs represent promising minimally invasive biomarkers for asthma endotyping and treatment monitoring. Therefore, collecting EVs could noninvasively inform the disease status of the cells, serving as a “liquid biopsy” (108), and EVs could open up new horizons for a better understanding of asthma pathomechanism and new targets for precision therapies (109). According to a recent review (110), EVs play a significant role in reduction in TNF-a secretion, TGF-*β* and IL-6 from primary alveolar macrophages, as well as upregulation of IL-10 secretion from peripheral blood mononuclear cells and promotion of proliferation and immunosuppressive capacity of Treg cells. EVs released from airway epithelial and immune cells, including eosinophils, mast cells, dendritic cells, macrophages, neutrophils and lymphocytes, can regulate immune cell activation, promote T2 inflammation, enhance eosinophil recruitment, and contribute to airway remodeling, thus influencing asthma and other atopic disease pathogenesis (111). Beyond their potential as liquid biopsy biomarkers, EVs are increasingly recognized as active regulators of asthma pathobiology. Engineered EVs are also being explored as therapeutic delivery systems capable of transporting anti-inflammatory miRNAs, siRNAs and other biologically active molecules directly to target tissues, although these approaches remain at the preclinical stage. Integration of EV-derived molecular signatures with established clinical biomarkers may improve identification of treatable traits and facilitate precision treatment selection.

Recent advances in multi-omics integration have highlighted the pivotal role of epigenetic modifications in asthma, revealing complex interactions between genetic predisposition, environmental exposures, and downstream molecular pathways that shape disease phenotypes (112). Established biomarkers, including blood eosinophils, fractional exhaled nitric oxide (FeNO), total IgE, and sputum eosinophils, remain the cornerstone of inflammatory endotyping and treatment selection in asthma. Rather than replacing these validated biomarkers, epigenetic signatures are more likely to provide complementary information by improving molecular endotyping, identifying novel treatable traits, and refining prediction of treatment response. Integration of epigenetic biomarkers with established clinical biomarkers and other omics platforms may further enhance precision asthma management (113). In addition, the convergence of multi-omics methodologies and machine learning approaches offers a powerful framework for delineating the molecular profiles of asthma endotypes while facilitating the discovery and prioritization of therapeutic targets through the integration of diverse biological data layers (112). Ultimately, the greatest clinical value of epigenetic profiling is likely to arise from its integration with other layers of biological information, including genomics, transcriptomics, proteomics, metabolomics, and established clinical biomarkers. Such multi-dimensional approaches may improve molecular endotyping, predict therapeutic response, and facilitate truly personalized asthma management.

## 5 Conclusions

Epigenetic mechanisms provide a mechanistic link between genetic susceptibility, environmental exposure, and clinically observable asthma traits. DNA methylation, histone modifications, and non-coding RNAs contribute to the regulation of type 2 inflammation, corticosteroid responsiveness, airway remodeling, and exposure-driven phenotypes, which are core components of the treatable traits framework. Although epigenetic biomarkers have not yet entered routine clinical practice, accumulating evidence suggests they may substantially refine asthma endotyping, improve prediction of treatment response, and facilitate implementation of precision medicine. Their greatest future value is likely to emerge through integration with established clinical biomarkers, imaging, physiological measurements, and multi-omics approaches rather than as standalone diagnostic tools. The use of minimally invasive tissues, such as the nasal epithelium and saliva, further enhances translational feasibility. Future progress will depend on longitudinal validation, cell-specific resolution, and integration with multi-omics platforms. If these challenges are addressed, epigenetic profiling may evolve from a descriptive tool into a clinically actionable component of precision asthma management.
